# Recovery and Survival of Patients After Out-of-Hospital Cardiac Arrest: A Literature Review Showcasing the Big Picture of Intensive Care Unit-Related Factors

**DOI:** 10.7759/cureus.54827

**Published:** 2024-02-24

**Authors:** Srdjan S Nikolovski, Aleksandra D Lazic, Zoran Z Fiser, Ivana A Obradovic, Jelena Z Tijanic, Violetta Raffay

**Affiliations:** 1 Pathology and Laboratory Medicine, Cardiovascular Research Institute, Loyola University Chicago Health Science Campus, Maywood, USA; 2 Emergency Medicine, Serbian Resuscitation Council, Novi Sad, SRB; 3 Emergency Center, Clinical Center of Vojvodina, Novi Sad, SRB; 4 Emergency Medicine, Department of Emergency Medicine, Novi Sad, SRB; 5 Anesthesiology, Resuscitation, and Intensive Care, Sveti Vračevi Hospital, Bijeljina, BIH; 6 Emergency Medicine, Municipal Institute of Emergency Medicine, Kragujevac, SRB; 7 School of Medicine, European University Cyprus, Nicosia, CYP

**Keywords:** coronary revascularization, cardiopulmonary ventilation, mechanical ventilation, targeted temperature management, intensive care unit, post-cardiac arrest, out of hospital cardiac arrest

## Abstract

As an important public health issue, out-of-hospital cardiac arrest (OHCA) requires several stages of high quality medical care, both on-field and after hospital admission. Post-cardiac arrest shock can lead to severe neurological injury, resulting in poor recovery outcome and increased risk of death. These characteristics make this condition one of the most important issues to deal with in post-OHCA patients hospitalized in intensive care units (ICUs). Also, the majority of initial post-resuscitation survivors have underlying coronary diseases making revascularization procedure another crucial step in early management of these patients. Besides keeping myocardial blood flow at a satisfactory level, other tissues must not be neglected as well, and maintaining mean arterial pressure within optimal range is also preferable. All these procedures can be simplified to a certain level along with using targeted temperature management methods in order to decrease metabolic demands in ICU-hospitalized post-OHCA patients. Additionally, withdrawal of life-sustaining therapy as a controversial ethical topic is under constant re-evaluation due to its possible influence on overall mortality rates in patients initially surviving OHCA. Focusing on all of these important points in process of managing ICU patients is an imperative towards better survival and complete recovery rates.

## Introduction and background

In the emergency medicine field of treating cardiac arrest (CA), the majority of efforts are being made to improve pre-hospital treatment of out-of-hospital cardiac arrest (OHCA), which is an important issue in the field of public health with several thousands of publications during the last 40 years [1]. CA has an incidence of 55 per 100,000 person-years and a low survival rate at hospital discharge [2-4]. However, managing CA patients involves more than just addressing pre-hospital treatment, as it is only the beginning stage that affects the outcome and eventual survival.

A significant percentage of patients who survive this condition end up in intensive care units (ICUs), regardless of CA cause. Therefore, hospitalization and ICU management following the occurrence of return of spontaneous circulation (ROSC) is the next link significantly influencing the outcome of these patients. As a matter of fact, studies very often investigate survival during hospitalization as a parameter of positive outcome after ROSC following both OHCA and in-hospital CA. With regard to that, there is an increasing need for improvement of ICU capacities and conditions for treating these patients, development of specialized departments, as well as focusing on specific aspects of recovery, with a special accent on post-CA shock and neurological injury (Table 1).

**Table 1:** Goals and tools of essential management strategies for improving survival rate in cardiac arrest survivors ROSC: return of spontaneous circulation; IV: intravenous; TTM: targeted temperature management; PCI: percutaneous coronary intervention; PaO2: partial pressure of oxygen; SaO2: oxygen saturation; PaCO2: partial pressure of carbon dioxide; MV: mechanical ventilation; WLST-N: withdrawal of life-sustaining therapy based on perceived poor neurological prognosis; CT: computerized tomography; MRI: magnetic resonance imaging; NSE: neuron-specific enolase; NFL: neurofilament light chain; S100-B: S100 calcium-binding protein B; EEG: electroencephalography; SSEP: somatosensory evoked potentials; ECMO: extracorporeal membrane oxygenation; ED: emergency department; MAP: mean arterial pressure

Strategy	Goals	Tools
Hemodynamic support	Keeping atrial pressure between 8 and 13 mmHg in the first 24 hours post ROSC, Keeping MAP above 65 mmHg	Rapid infusion of large volume, ice-cold IV fluids, Vasopressors, Inotropes
Coronary reperfusion (in post-myocardial infarction cardiac arrest survivors)	TTM recanalization of coronary arteries	Rapid infusion of large volume, ice-cold IV fluids, PCI
Adequate oxygenation	Keeping PaO2 between 60 and 200 mmHg, Keeping SaO2 higher than 90-92%, Keeping PaCO2 between 35 and 45 mmHg, Preventing delayed extubation	Mechanical ventilation, Treating flail chest, Treating pneumonia, Bronchoscopy
Protection of diaphragm	Myotrauma prevention	Titrating MV, Diaphragm thickness measurement using point-of-care ultrasound
Targeted temperature management	Keeping body temperature between 32°C and 36°C	Rapid infusion of large volume, ice-cold IV fluids, Wet blankets, Ice packs
Neuroprognostication	Regaining consciousness, Reducing ischemia and ischemia-reperfusion injury, Preventing WLST-N	Daily clinical examination, Neuroimaging (CT, MRI), Biomarkers measurement (NSE, NFL, S100-B), Electrophysiology (EEG, SSEP)
Extracorporeal membrane oxygenation	Keeping optimal blood oxygenation Improving survival rate	Installing ECMO within 10 minutes after arrival at the ED

## Review

Post-CA syndrome and post-CA shock

The term post-CA syndrome refers to the presence of the following four elements: neurological injury, myocardial dysfunction, systemic ischemia-reperfusion injury, and multi-organ failure [5]. Since CA as an event can lead to significant neuronal damage and impaired function of other vital organs, management of CA survivors in ICUs is a factor of paramount importance for their successful recovery in the post-resuscitation period.

Post-CA shock occurs in approximately 68% of OHCA patients during their ICU stay [6] and can interact with pre-arrest hypertensive disease, especially in terms of influencing neurological outcomes [7-9]. Post-CA shock patients are more frequently females and present with non-shockable initial heart rhythm. Also, the time from collapse to ROSC is longer, while arterial lactate and blood creatinine values at admission are higher in patients having post-CA shock. However, OHCA cause is usually not significantly associated with post-CA shock occurrence [6].

Post-OHCA hospital and ICU survival rates and mortality

European Registry of Cardiac Arrest (EuReCa) project reported a hospital survival rate of approximately a quarter of all OHCA patients in the European region [10] and there is a great diversity of findings by many worldwide studies.

Higher rates of survival are observed among OHCA patients where collapse was witnessed, in comparison with non-witnessed events. Also, among bystander-witnessed collapses, higher survival rates are also noted in cases where bystander-provided cardiopulmonary resuscitation (CPR) measures, in contrast to patients where bystanders did not initiate CPR [4].

Chronic kidney disease, chronic heart and respiratory failure, liver cirrhosis, diabetes mellitus, malignancies, and hematologic diseases are all strongly associated with higher hospital mortality rates of CA patients. In the case of all these comorbidities, there is up to three times greater chance for a fatal outcome during hospitalization [11].

During ICU stay, 47-66% of post-OHCA patients die [6,11-16]. Considering patient-related, pre-hospitalization, and hospital admission-related factors, survivors are usually younger patients [6,17], with a cardiac cause of OHCA [6], shorter duration of CPR measures application [6], higher rate of bystander CPR [6], higher frequency of shockable initial hearth rhythm [6], as well as higher albumin levels [17] and lower lactate and creatinine values [6] on admission. Independent factors associated with higher ICU mortality rates are longer collapse-to-ROSC time [6], patient age [6,17], and higher lactate value on emergency ward admission [6]. Most patients die from withdrawal of life-sustaining therapy (WLST) six days following ICU admission [6]. Among those who die earlier, brain death or poor neurological prognosis are the most common findings [6].

Low systolic arterial blood pressure within one hour of admission to the ICU has been observed as an independent predictor of in-hospital mortality [9,18]. Shock and multi-organ failure have been presented as causes of approximately one-third of death outcomes in OHCA patients [6,19] and three modes of death have been reported so far: brain trauma (71% of deaths), shock (16% of deaths), and miscellaneous factors (13% of deaths) [16].

Additionally, one of the largest studies performed on post-CA patients published to date reported that during hospitalization, post-CA shock as the main cause of death in ICU patients in the first three to four days of hospitalization is usually replaced with neurological injury as the main mode of death from day 4 of ICU hospitalization. Furthermore, the same study observed age, collapse-to-ROSC time, and admission arterial lactate values as risk factors for high mortality rates in post-CA shock patients, while shockability of initial heart rhythm, collapse-to-ROSC time, and arterial lactate values on admission as risk factors for death related to neurological injury [6].

Organ perfusion

Considering hemodynamics, in order to maintain adequate end-organ perfusion, blood pressure needs to be at a reasonable level, which is usually achieved by using vasoactive agents. On the other side, there is a need for minimizing the afterload of the left ventricle (LV) and decreasing mesenteric/peripheral vasopressor-related ischemia. The multifactorial nature of arterial hypotension after OHCA is a major challenge. The main factors influencing the drop in arterial blood pressure are post-resuscitation shock (as a consequence of ischemia-reperfusion organ injury), cardiogenic shock, and underlying cardiac pathology [20]. Persistent hypotension is associated with worse neurological recovery and multiple organ failure, leading to higher mortality rates after OHCA [6,21,22].

There is a low cardiac output after CA [23], as well as fluid extravasation, resulting in a reduction in colloid osmotic pressure of interstitial space [24,25]. Additionally, autoregulation of cerebral blood flow is disturbed because of brain damage, as well as disturbed cerebral perfusion pressure and mean arterial pressure (MAP) [26]. Thus, increasing MAP might increase cerebral perfusion, and targeting a specific MAP level is feasible in comatose patients after CA [27].

Rapid infusion of large-volume, ice-cold intravenous (IV) fluid is an effective procedure for increasing blood volume and inducing therapeutic hypothermia. Although LV function is reduced in these situations, concomitant volume expansion does not cause further disturbance in respiratory function after CA [28-33]. Approximately 3.5-6.5 L of IV crystalloid is required in the first 24 hours after ROSC to maintain 8-13 mmHg of atrial pressure [21,23,34].

Besides the hemodynamic support rapid infusion of large volumes of ice-cold IV fluid offers [21], it also induces mild therapeutic hypothermia but does not have the potential to maintain it for 24 hours [32]. Therefore, to keep body temperature within the range of 32-34°C, special external or internal cooling devices with continuous temperature feedback can be used [21,35-37]; less expensive methods such as cold, wet blankets or ice packs combined with ice-cold fluids [21,38] can also be used, but those alternatives could result in greater temperature fluctuations [21,39].

In comatose survivors of CA receiving post-resuscitation care (percutaneous coronary intervention, inotropes, vasopressors, and/or mechanical ventilation (MV)), rapid ice-cold IV fluid infusion to increase intravascular volume and achieve a target temperature of 32-34°C had a partially positive effect on cardiorespiratory function by being associated with a slight worsening of oxygenation, but also a preservation of myocardial function and subsequent improvement of LV function [33]. With applied therapeutic hypothermia and cardiovascular support, comatose ventilated CA survivors require and tolerate large volumes of IV fluids in the first few days after ROSC [24,33,40].

Severe vasoplegia and vasodilation in post-resuscitation shock also require hemodynamic management with vasopressors and inotropes, especially when post-resuscitation myocardial dysfunction is present. Norepinephrine is most commonly used as the first-line vasopressor because it does not aggravate the arrhythmogenic effects of other catecholamines. Dobutamine is the most established inotrope in this situation with the most effective dose of 5 µg/kg/min [41,42].

Higher MAP is associated with better outcomes, with a threshold of 70-75 mmHg [43,44]. There is a possibility that the optimal MAP may differ between patients and there is a need to individually estimate the optimal MAP [7]. In post-CA patients with shock after AMI, targeting a MAP between 80 and 100 mmHg with additional inotropes and vasopressors during the first 36 hours of ICU stay is associated with less myocardial injury [45]. During the 36 hours of the interventional period with norepinephrine, dobutamine, and blood transfusions, patients have higher MAP, while cerebral perfusion and oxygenation during the first 12 hours are significantly higher [46].

High doses of vasoactive agents are, on the other side, also associated with negative outcomes [47-49]. Therefore, MAP of 65 mmHg (or higher in case of significant vasculopathy), as an optimal blood pressure target, is commonly set as a goal typically requiring a balance of IV fluid administration and inotropic and/or vasopressor support [50].

Although cerebral oxygenation is unaffected by MAP [27,51], the use of vasopressors and inotropes can have some downsides, mostly related to myocardial function. For example, myocardial damage caused by acute coronary syndrome and myocardial ischemia-reperfusion injury is common after CA and CPR. Therefore, excessive vasopressor use may lead to increased afterload, contractility, heart rate, and stroke work, resulting in an unfavorable increase in myocardial oxygen consumption, thereby aggravating myocardial damage [52]. On the other hand, hypotension or even low-normal blood pressure at hospital admission can decrease coronary perfusion pressure leading to myocardial hypoperfusion and ischemia and, eventually, cardiovascular collapse, leading to increased mortality risk in patients with acute myocardial infarction (AMI) [53,54], although some studies showing that there is no association between MAP and the extent of myocardial damage [27].

Also, theoretically, targeting a higher MAP using additional fluid volume, vasopressors, and inotropes may result in pulmonary edema, limb ischemia, cardiac arrhythmias, and re-arrest in vulnerable patients, such as CA patients with a recent AMI and depressed LV function [46].

Acute kidney injury (AKI)

AKI has been registered in up to 80% of OHCA survivors of which one-third require continuous renal replacement therapy [55,56]. It has been associated not only with ischemia but also with ischemia-reperfusion kidney injury, leading to prolonged loss of renal autoregulation [57,58]. This is one of the reasons why duration of resuscitation measures, along with age, gender, collapse occurring in a public setting, initial heart rhythm, and post-resuscitation syndrome have been previously characterized as the factors increasing the risk for developing severe forms of AKI in patients initially surviving OHCA, further raising the risk for progression to chronic kidney disease, poor neurological outcome, and death [49,59-61]. Also, severe AKI has a significant impact on treatment costs as well as on both short-term and long-term morbidity and mortality [55,59,60].

High MAP levels can significantly contribute to decreasing the need for continuous renal replacement therapy in OHCA survivors [62]. In patients who undergo targeted temperature management (TTM) treatment methods, the percentage of time spent with MAP lower than 85 mmHg during the first 12 hours following ICU admission is independently associated with an increase in the risk for developing severe AKI forms. The association is even higher during the first six hours after admission [20]. However, the use of MAP seems to lack both sensitivity and specificity in predicting severe forms of AKI [63]. Additionally, the association between the severity of arterial hypotension and the occurrence of severe forms of AKI has also been confirmed [20]. This evidence emphasizes the benefit of early targeting of high MAP levels to prevent kidney injury in OHCA survivors.

ICU-related factors in post-OHCA patients and initial post-ROSC management

Some of the most important ICU factors influencing the overall survival rate of initial CA survivors are ventilatory support or MV, enteral/parenteral nutrition, renal support, frequent dressing changes, application of diuretics and/or vasoactive medications, insertion of peripheral arterial catheter, and appropriate care of drains [11].

There is evidence that post-CA care, specifically hemodynamic, respiratory, and metabolic optimization, are all connected with improved survival and minimization of neurological sequelae. The two most important steps in the implementation of post-resuscitation measures are application of TTM as well as early invasive coronary evaluation with revascularization procedure as an appropriate method of management at CA care centers [64-67]. Monitoring of hemodynamic parameters and cerebral function during the post-ROSC period is also very helpful since it allows early and continuing detection of abnormalities with simultaneous administration of post-CA care measures [50].

Urgent coronary reperfusion

As a common precipitating CA cause, coronary ischemia is still under-addressed and is evident in 30-84% of CA patients [66,68,69]. In some cases, additional health problems can further contribute to the pathophysiology complexity [70]. Several observational studies showed favorable survival and neurological function in CA patients having presumed ischemic etiology and who are undergoing urgent percutaneous coronary intervention along with concurrent cooling through applying TTM measures which do not delay revascularization procedure [71-74]. Despite the fact that post-ROSC electrocardiogram findings may not show a significant correlation level with the degree and coronary obstruction site, a 12-lead electrocardiographic measurement should still be obtained to determine whether ST elevation is present [66,72,75]. Similarly, post-ROSC cardiac biomarkers, with the exception of markedly elevated troponin levels, have low sensitivity and specificity for predicting acute occlusive lesions in coronary vessels [66,75].

In cases of significant occlusion of coronary arteries associated with acute coronary syndrome, even cooled CA patients should undergo a revascularization procedure [66,75], with aspirin and heparin being administered before catheterization. Also, there is a need to consider fibrinolysis when a revascularization delay of at least 12 hours is expected [75,76]. In case when percutaneous coronary intervention is necessary, it is recommended to manage patients with dual anti-platelet therapy following stenting with aspirin and another P2Y12 receptor inhibitor [77-80]. Additionally, maintaining MAP levels between 80 and 100 mmHg can decrease the chances of myocardial injury in post-CA patients with acute myocardial dysfunction followed by shock [47].

As opposed to the majority of findings, some studies, however, observed no association of early cardiac catheterization use with lower mortality and permanent neurological sequelae, but only considering other baseline risk factors with an influence on survival and recovery [81].

Mechanical ventilation issues in ICU OHCA survivors

Compared with normoxemia, hyperoxemia (PaO2 > 200 mmHg) and hypoxemia (PaO2 < 60 mmHg) in post-CA patients are associated with increased mortality rates [82,83]. Hyperoxemia can potentiate tissue injury, presumably via direct oxygen toxicity or indirectly, by forming oxygen-free radicals [21]. While hyperventilation-induced hypocapnia (PaCO2 < 35 mmHg) portends worse outcomes in a post-CA setting, hypoventilation-induced hypercapnia (PaCO2 > 45 mmHg) may or may not affect outcomes [82,83]. Thus, FiO2 should be titrated to keep PaO2 to 60-200 mmHg and SaO2 > 90-92%, and MV to keep PaCO2 to 35-45 mmHg [66].

There are suggestions that useful ways of rational diaphragm-protective MV strategy are careful ventilator settings titration and focusing on airway pressure waveform contour, all in order to avoid negative effects of both excessive and insufficient inspiratory effort [84,85]. With this in regard, some preventive strategies may be implemented to tailor MV assistance and keep the diaphragm function stable during the ventilation period. Also, use of diaphragm pacing as a promising innovative approach could also enhance prognosis in the context of these patients’ recovery [86]. Monitoring levels of respiratory muscle effort along with adjusting ventilation should, therefore, be considered by clinicians from the very beginning of the MV procedure in these patients to ensure lung protection, satisfactory gas exchange, appropriate level of inspiratory effort, and patient comfort [85].

Predicting optimal extubation time is also a challenge, especially in patients with underlying diaphragm dysfunction. A significant contribution in identifying patients with normal function of the diaphragm and decreasing time to extubation can be provided by incorporating ultrasound assessment of diaphragm function into usual care [87]. An open-label randomized controlled trial published in 2021 tested and compared both preventive and curative strategies for fluid removal to shorten the duration of weaning from MV and eventually improve the outcomes of patients on MV [88].

Delayed extubation (DE) can be a result of several factors. Female sex, advanced age, and flail chest could be risk factors for DE. Flail chest due to multiple rib fractures, and especially fragility fractures after chest compressions for CA occur more frequently in women or elderly individuals with osteoporosis [89-91]. In addition, chest compressions may lead to lung injury [92]. Also, persistent unconsciousness can further increase the risk of DE in CA survivors [91].

Additionally, if there is an underlying pulmonary disease with a poor respiratory function, exposure to high oxygen concentrations, MV-induced barotrauma, and MV-associated pneumonia after CPR may increase the risk for further worsening of already poor respiratory function resulting in MV dependence and DE [91,93,94].

Pneumonia is frequently found after post-CA syndrome (PCAS), which can deteriorate the ventilatory situation. Pneumonia is common during ICU stay with a subsequent impact on morbidity and mortality [95-98]. Within the first days of post-ROSC hospitalization, early-onset pneumonia can be present in up to 65% of patients [95,96,98]. Some of the mechanisms and risk factors for this complication are loss of natural airway clearance, emergency intubation with possible aspiration, lung contusion, coma, and MV [99-101].

An independent risk factor for early-onset pneumonia, in addition to aspiration, is therapeutic hypothermia [95,98,101-104]. The impact on survival and neurological outcome is minimal in early-onset pneumonia [96]. However, infections following OHCA, and especially the incidence of late-onset pneumonia are associated with prolonged MV time and ICU stay [100,105]. Prophylactic antibiotic therapy could be a good option for preventing this complication but has shown no effect on MV duration, length of ICU stay, mortality, and neurological outcome [96,98].

Also, after admission to the ICU for post-CA care, bronchoscopy is a usual procedure to improve impaired ventilation and as a diagnostic endobronchial lavage for microbiological analysis before starting antibiotic treatment. Bronchoscopy performed within 48 hours after hospital admission is associated with higher rates of MV-free patients, emphasizing its importance in the management of PCAS patients. Therefore, early bronchoscopy may have a beneficial effect on patients after OHCA in terms of duration of intubation and invasive MV [106].

The role of diaphragm function assessment and point-of-care ultrasound in ICU patients

Additionally, diaphragm dysfunction is an important clinical concern in critically ill patients and an expanding body of evidence shows that diaphragm weakness is frequent since its force is influenced by many different ICU-related factors [86,107]. Furthermore, in ventilated patients, diaphragm weakness is even more common through multiple mechanisms, referred to as myotrauma. It prolongs ventilator dependence and contributes to increasing mortality rates [108].

Therefore, diaphragm function in OHCA survivors has significant importance, with diaphragm ultrasonography as a promising tool for monitoring its activity [89]. Preventing diaphragm myotrauma is now recognized as a priority in patients on MV support and it has potential to significantly improve outcomes in these patients [85].

Significant progress has already been made in the assessment of diaphragm function at the bedside with ultrasonography methods playing an important role. The accessibility of ultrasound use in these patients is, therefore, a dominant question that needs to be addressed, since it may help recognize and manage diaphragm dysfunction in a more advanced way during weaning from MV support [86].

The usefulness of monitoring diaphragm function in ICU reflects in providing help to physicians in a more profound understanding of the interaction between patient’s breathing effort and ventilator support, thus facilitating the decision-making process in setting MV parameters. Ultimately, monitoring of diaphragm function has its special use in patients with difficult and prolonged weaning from MV by predicting weaning failure and diagnosing diaphragm dysfunction [109].

With regard to myotrauma, keeping inspiratory effort during MV measures application within the adequate value limits reduces the amount of injury and expresses the potential to improve outcomes in post-OHCA patients. Patient-ventilator dyssynchrony may have a negative impact on diaphragm function which can be managed by selecting some proportional assist modes of ventilator in order to reduce dyssynchrony. Monitoring respiratory effort during MV is also an important element of protective MV measures [108]. This implies that its great importance lies in the right balance between lung protective MV strategy and maintaining diaphragm activity [107].

Diaphragm thickness is also another important point studied in the last few years. A study published in 2014 showed that ultrasound measures of diaphragm muscle thickening may predict extubation success. This measure of diaphragm function can be performed at the bedside and requires no special effort by the patient. The ubiquitous presence of ultrasound equipment in ICUs is an additional facilitating factor. The portability and availability of ultrasound make measuring diaphragm thickness ideally suited for incorporation into the intensivist’s decision-making process of evaluating extubation outcomes [110]. Another study described important changes in diaphragm muscle thickness that may be caused by excessive or inadequate ventilatory support that may contribute to the development of ventilator-induced diaphragm dysfunction. These changes in diaphragm configuration might be prevented by muscle-protective MV strategies titrated to optimize patient inspiratory effort [111].

A longitudinal cohort study published in 2015 analyzed the evolution of ultrasound-measured diaphragm atrophy in ventilator-induced dysfunction of diaphragm in patients hospitalized in ICUs. That study described duration of MV as an additional factor associated with the degree of diaphragm atrophy, along with other already known muscle atrophy risk factors. Diaphragm atrophy showed fast progression, with the highest decrease in diaphragm thickness occurring during the first 72 hours of ventilation support [112].

TTM

TTM represents a strategy of deliberate active cooling, rewarming, and extended fever control [95]. Post-ROSC, all comatose OHCA survivors older than 18 years of age are recommended to undergo temperature management between 32°C and 36°C [39,113-115]. Cooling and active pyrexia prevention lowers end-organ demands, including brain demand, by decreasing metabolic needs of tissues. With this procedure, infarction size could be potentially limited, and even more importantly, ischemia-reperfusion injury could be attenuated [21]. The absence of effective TTM may lead to the occurrence of fever above 37.7°C which has been proven to be associated with poor outcomes, with the worst outcomes in cases with a body temperature above 39°C [66].

Application of TTM measures contributes to reducing end-organ CO_2_ production, further lowering MV requirements and enabling the use of lower tidal volumes (6-8 ml/kg) towards achieving normocapnia. This represents a very important point since an expanding body of literature supports low tidal volumes and plateau pressures lower than 30 cmH_2_O, including the cases without acute respiratory distress syndrome since high volumes and pressures have been proven to cause or contribute to the initiation of MV-induced lung injury in critically ill patients [114]. The Targeted Therapeutic Mild Hypercapnia After Resuscitated Cardiac Arrest (TAME) trial is one of the rarest studies advancing the understanding of this issue by separating normocapnia (PaCO_2_ 35-45 mmHg) and mild hypercapnia (PaCO_2_ 50-55 mmHg) as important points during the 24 hours of post-arrest period [113]. Furthermore, there are findings that no significant difference exists in TTM normothermia vs. hypothermia [116]. Certainly, this topic requires further investigation.

TTM, like all other measures implemented during the early post-CA hospital care, is not flawless and can certainly result in some side effects, such as shivering. Therefore, typical components of applying TTM measures during early post-CA care should be the application of continuous intravenous infusions of sedative-hypnotics and analgesia, along with neuromuscular blockade. Sedation should be always monitored by using some of the widely accepted sedation scales or bi-spectral index and spectral edge frequency monitoring, if available [66].

Neurological recovery

After survival rate, neurological recovery is the next most important outcome in patients with CA and is dichotomized as favorable and poor [117]. Besides, post-resuscitation circulatory failure (caused by systemic ischemia-reperfusion) and post-anoxic brain injury are some of the main factors leading to early mortality in hospitalized OHCA survivors [6].

In CA survivors, neurological outcomes are better in males [118], patients having prehospital ROSC [119], patients undergoing TTM [28,39], and patients who received extracorporeal CPR [120]. Also, inter-facility transfers can impair neurological recovery by increasing the probability of clinical deterioration in initial CA survivors [121].

The number one cause of poor long-term outcomes and mortality after OHCA has proven to be hypoxic-ischemic encephalopathy (HIE) [16,122,123]. Although the advancement of resuscitation measures application is evident, the majority of resuscitated OHCA survivors present with altered consciousness levels due to irreversible HIE [6,16]. Two-hit model divide hypoxic-ischemic brain injury into primary and secondary phases which occur during CA and immediately after ROSC, respectively [124].

Additionally, low systemic vascular resistance and myocardial dysfunction have high potency in initiating circulatory shock during the early phase after CA with an incidence of 15-68% [6,95,125-127]. This type of shock is usually defined as a combination of hypotension, and signs of hypo-perfusion, along with a need for measures of maintaining adequate perfusion pressure. The importance of circulatory shock in the post-CA period lies in its potential to aggravate HIE by prolonging cerebral hypo-perfusion [9].

The severity of hypotension in OHCA survivors with MAP below 75 mmHg during the first 96 hours after admission is associated with increased rates of severe neurological dysfunction [45], and maintaining MAP levels above 90 mm Hg within the first six hours after admission ensures high chances of good neurological outcome at hospital discharge [8]. On the other side, some studies showed no association between neurological outcomes and MAP levels [27].

Although it is overrepresented in patients having pre-existing arterial hypertension, impairment of cerebral perfusion autoregulation in 35% of post-resuscitation cases [7] and current recommendations suggest maintaining MAP above 65 mm Hg during the initial post-resuscitation care [7,18,35,45,128-141]. With regard to this, some recent studies suggest that circulatory shock is an independent predictor of poor neurological outcomes at hospital discharge [9], while others do not support that finding [128].

Although there is always a question of whether coronary revascularization should be performed before or after the steps to ensure neurological recovery, the fact is that there is always a small chance for neurological recovery to be reliably determined before or at the time of emergency coronary assessment in post-CA patients, despite some public reports of post-procedural deaths which created an impetus to avoid emergency coronary angiography in comatose patients. Studies showed that there is a higher mortality rate in these situations, mainly due to poor neurological recovery. This implies that the potential benefits of emergency coronary revascularizing intervention and placement of temporary support devices as a part of early catheterization strategy should not be withheld, except for cases where the neurological prognosis is clear or can be easily estimated at the time of presentation [50].

Physiological, pharmacological, and surgical methods are the essential neuroprotective strategies for managing secondary brain injury. These methods, however, may limit the improvement of outcomes, since primary brain injury remains the main factor contributing to the occurrence of advanced brain injury before neuroprotective measures are applied [142,143]. Therefore, neuroprotective strategies, such as modulation of physiological factors, which can lead to suppressing secondary brain injury, can be applied in post-CA care without assessing the severity of primary brain injury [48,144,145].

Neurologic prognostication following ROSC may be conducted at any moment during patient management, including out-of-hospital, ED, and ICU environments [146]. Clinical neurological examination is the foundation of this process and is supported by electroencephalography (EEG), somatosensory evoked potentials (SSEP), neuroradiological imaging, and biomarker analysis [147].

About 80% of OHCA survivors admitted to an ICU are comatose [148] and two-thirds of them will die due to a hypoxic-ischemic brain injury (HIBI) [16,19]. However, most of these deaths result from WLST following prognostication of a poor neurological outcome [149-151] which often occurs within the first day of admission [152]. Therefore, WLST remains the most common cause of in-hospital death for patients resuscitated from both IHCA and OHCA [16,150].

About one-third of deceased patients hospitalized after OHCA have WLST within 72 hours after admission because of a perceived poor neurological prognosis [152]. However, approximately 16-19% of PCAS patients die due to WLST within 72 hours post-CA, despite a predicted good neurological outcome [152-156]. Nevertheless, WLST is occasionally performed earlier than 72 hours after ROSC due to medical factors, patient values, and preferences, or premature neurological prognostication related to ICU admission [141,152,155,157,158].

Therefore, early and accurate prediction of neurological prognosis in CA survivors is important to appropriately distribute medical resources and to prevent premature WLST in patients with neurological recovery potential [159]. International guidelines for post-CA care recommend that neurological prognostication should be delayed at least 72 hours after ROSC [141,160].

**Figure 1:**
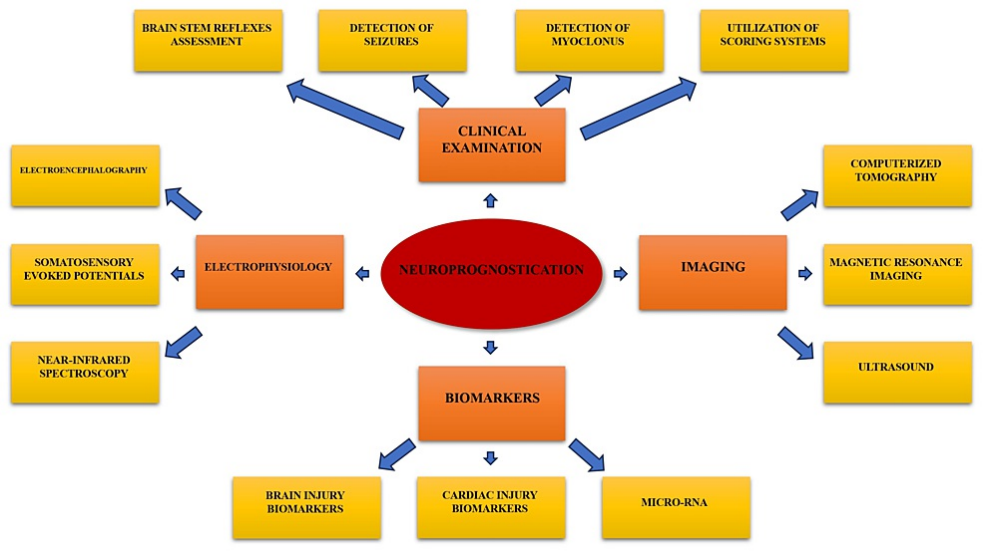
Strategy for assessment of neurological recovery in cardiac arrest survivors Image credit: Authors

Several approaches can be used in neuroprognostication in PCAS patients, including clinical examination, neuroimaging studies, electrophysiology, and measuring levels of certain biomarkers. A combination of these methods can be a very good comprehensive approach to predicting neurological outcomes in these cases. In fact, recommendations suggest a multimodal approach to assessing neurological conditions which should occur at least 72 hours after ROSC, including clinical examination, electrophysiology, serum biomarkers, and neuroimaging (Figure 1) [141,160-163].

Clinical Examination

Neurological assessments in comatose CA survivors are the basis for neurological prognostication [21,154,164,165]. Clinical signs that can be collected during a daily bedside neurological examination are crucial in neuroprognostication [166,167]. The assessment of consciousness is also important since approximately 50% of patients never regain consciousness or remain severely disabled as a result of HIBI [168,169].

Determination of the presence or absence of spontaneous breathing, cough reflex, gag reflex, pupillary response to light, corneal reflex, oculocephalic/oculovestibular reflexes, as well as heart rate variability can reveal the integrity of most of the cranial nerves and brainstem. From a prognostication standpoint, pupillary and corneal reflexes have traditionally and consistently been most valuable [154,164,170,171]. Heart rate variability is also evidence of brainstem integrity [167,172]. However, since the brainstem is the least susceptible central nervous system region to injury from anoxia, many patients, even those with poor outcomes, may have fully or partially intact brainstem function; however, when dysfunction is present, it likely reflects more severe brain injury [167].

Seizures may also be related to a worse prognosis [173] but may respond readily to treatment with antiseizure medications, thereby allowing recovery to a good neurological state [174]. EEG monitoring is usually necessary for detecting seizures, especially in newborns and infants [175,176].

Additionally, there are various findings in the literature debating regarding the prognostic potential of myoclonus for poor neurological outcomes [152,170,177-184]. There are various findings of myoclonus as a predictor of post-CA neurological outcome and its use is recommended only in combination with other indices [162], particularly EEG [185,186].

There are several scoring systems as clinician-reported measures of neurological function after CA which are used in clinical practice to predict neurological outcome [146,187-209], with Cerebral Performance Categories score [187], Cerebral Performance Categories - Extended score [188], Glasgow Outcome Scale - Extended score [189], and Modified Rankin Scale [190] being used more frequently compared to others (Table 2).

**Table 2:** Neuroprognostication scoring systems used in cardiac arrest survivors OHCA: out-of-hospital cardiac arrest; TTM: targeted temperature management; ROSC: return of spontaneous circulation

Score	Reference Number
Cerebral Performance Categories score	187
Cerebral Performance Categories - Extended score	188
Glasgow Outcome Scale - Extended score	189
Modified Rankin Scale	190
Brainstem Reflex Score	191
Cardiac Arrest Hospital Prognosis score	192, 193
C-GRApH score (coronary artery disease known pre-OHCA, glucose ≥ 200 mg/dL, rhythm of arrest non-shockable, age > 45 years, pH of arterial blood ≤ 7.0)	194, 195
OHCA score	196
SALTED (shockable rhythm, age, lactate, time elapsed until ROSC, and diabetes) score	197
Cardiac Arrest Survival Score	198
Brain Death After Cardiac Arrest score	199
Post-cardiac arrest syndrome for therapeutic hypothermia (CAST) score	200-202
SLANT score	193
MIRACLE2 risk score	146, 195, 203-209
TTM-risk score (used in TTM-treated OHCA survivors)	195

Neuroimaging Studies

Several studies have aimed to predict neurological outcomes during the early stage (i.e., before TTM) using neuroimaging examinations, such as CT and MRI [158,210-213].

Brain CT is often performed early in the admission but is typically not helpful for prognostication until several days after CA [214-219]. Loss of gray-white matter ratio is indicative of cerebral edema and correlates with hypoxic-ischemic changes in different areas of the brain [220,221]. Cerebral edema is the main CT finding of HIBI following CA, occurs early after CA, and can be a good predictor of poor neurological outcome [210,222-225].

HIBI after CA appears on brain MRI as hyperintense areas on diffusion-weighted imaging. Determining the whole brain-apparent diffusion coefficient can be used for determining the prediction of poor neurological outcomes after CA [210,226-229]. Brain MRI is especially useful two to five days after ROSC [230] but can predict neurological outcomes even only three hours after ROSC [210,231].

Functional MRI constitutes a complementary diagnostic tool during early-stage (less than 72 hours) post-CA coma to support clinical decisions after ROSC and can be a good predictor of long-term neurological outcome in OHCA survivors if performed within 48 hours after the onset of HIBI following ROSC [232].

Optic nerve sheath diameter measured ultrasonographically at least six hours following ROSC can also serve as a predictor of poor neurologic outcome after OHCA [233]. However, immediate post-ROSC measurement does not correlate with subsequent measurements made at 24-, 48-, and 72-hour intervals, nor with patient outcome [234].

Biomarkers

In recent years, a number of biomarkers have emerged, which potentially could improve current algorithms for the prediction of neurological outcomes. Advanced neurologic serum biomarkers such as neuron-specific enolase, neurofilament light chain, and S100-B protein have been studied in an effort to predict outcomes for CA patients.

Neuron-specific enolase is released after neuronal injury [235-238], is currently the only biomarker included in the guidelines [147], and is the most widely available and best-documented biomarker of cerebral injury, especially among comatose CA survivors [239,240]. Another promising biomarker is the neurofilament light chain, released after axonal injury [241-243], which is especially useful for the discrimination of long-term neurological outcomes as early as 24 hours after ROSC [243,244].

Other biomarkers of brain injury, including S100 calcium-binding protein B (S100-B), tau protein, glial fibrillary acidic protein, and ubiquitin C-terminal hydrolase-L1, have also shown potential in CA prognostication [245-248]. S100-B is released after astroglial and Schwann cell injury [249-253] and is measured immediately after ROSC is significantly higher in those with a poor neurologic outcome [242].

Biomarkers of cardiac injury such as troponin T, N-terminal pro-B-type natriuretic peptide, and copeptin, along with biomarkers of systemic inflammation such as procalcitonin and interleukin-6 are also associated with neurological outcome [254-258].

Also, preliminary studies on micro-RNA indicate that miR-124-3p can be an independent predictor of both survival and neurological outcomes in comatose CA survivors [259].

Electrophysiology

EEG analyses have shown that measures of brain activity are highly sensitive to HIBI [168,260-262]. EEG can provide important prognostic information even when it is recorded within the first 24 hours after ROSC [263]. Malignant electrographic brain patterns, including burst suppression, low voltage, stimulus-induced discharges, and identical bursts all correlate with poor neurological outcomes [264-279]. Continuous or intermittent EEG evaluation may help in identifying epileptiform patterns and assessing the appearance of brain electrical activity waveforms [280]. In evaluating this method’s results, poor neurological outcomes in CA survivors are usually strongly predicted by wave suppression with or without discharges on EEG findings [281,282]. Very low chances of regaining consciousness in comatose patients occur if EEG patterns are unfavorable seven days after ROSC [118]. In the later course of recovery, neurological outcomes can be assessed using different measurement methods of neurological performance [283], but caution is always needed because even patients with a favorable recovery may have ongoing sub-clinical cognitive and/or neuropsychiatric sequelae, emphasizing the need for individualized treatment and rehabilitation strategies [284].

Also, multiple studies have evaluated the role of SSEP in predicting outcomes after CA. Absent cortical responses on SSEP are a robust tool for neuro-prognostication in PCAS patients [157] and suggest loss of integrity of thalamocortical projections (N20 potentials) correlates with poor neurological outcome [285-291]. The bilateral absence of the N20 cortical waves of SSEP at 72 hours from ROSC predicts a poor neurological outcome with high accuracy and precision [292]. An advantage of SSEP over EEG is that they are less affected by sedation. However, the usefulness of the presence of N20 response is limited due to low sensitivity and low positive predictive value [164,286,291,293] Also, SSEP analysis may be prone to electrical interference [15] and can be subject to noise interference and inter-observer variability [287].

Near-infrared spectroscopy is an additional method for neuroprognostication in PCAS patients, since altered cerebral blood flow is considered one of the mechanisms causing HIBI [125]. This technique is a non-invasive technique for monitoring regional cerebral oxygen saturation at the microvascular level, which can be an early (during the first 48 hours post-ROSC) predictor of six-month poor neurological outcome in PCAS patients, but with low accuracy (52% sensitivity and 55% specificity) [294].

Assessing the Function of Other Vital Organs

Up to 96% of patients resuscitated after CA demonstrate some degree of organ dysfunction, with two-thirds having at least two extracerebral organs involved [295]. Non-survivors have a greater incidence of renal, respiratory, and cardiovascular failure on admission than survivors. Similar patterns are seen in patients with unfavorable versus favorable neurological outcomes [296]. Therefore, assessment of vital organ function may be an additional method contributing to the overall neuroprognostication process in PCAS patients.

Extracorporeal membrane oxygenation (ECMO)

The use of ECMO has become increasingly prevalent in the management of patients with CA, especially in those having refractory CA and those who achieve ROSC and have ongoing hemodynamic instability, because it can improve survival and neurological outcomes in a subpopulation of patients with CA who would otherwise uniformly die [297-300].

Utilization of ECMO for OHCA has been steadily increasing each year but still represents only a small fraction of the affected population [301] with a larger utilization in patients with OHCA of presumed cardiac etiology with attempted resuscitation [302].

The use of ECMO for refractory OHCA has been associated with survival rates between 6.9% and 56.0% [303-308]. Initial shockable rhythm, shorter low-flow time, higher arterial pH on admission, higher arterial partial pressure of oxygen, lower arterial partial pressure of carbon dioxide, and lower serum lactate on admission are associated with favorable outcomes in OHCA patients on ECMO [309,310], while increased duration of CPR is associated with higher risk of brain death [310] and older age is a factor of lower survival rate [308].

In patients with refractory OHCA, the survival rate can be improved by mobilization of the ECMO team within 10 minutes for refractory OHCA, and rapid and accurate implantation of ECMO. Activation of the ECMO team within 10 minutes in patients having refractory CA (in ED or catheterization laboratory) may improve the 30-day survival rate to almost 50% [311].

ECMO has great potential in reversing signs of poor neurological outcomes (e.g., pupillary reflex). Additionally, specific factors, not otherwise significant, such as oliguria in the first 24 hours after ECMO, are highlighted as independent predictors of survival in patients on this type of treatment [311].

Once the patient is assisted and stabilized on ECMO, treatment of the suspected cause of CA should be initiated. This usually includes immediate coronary angiography with percutaneous coronary intervention, given the fact that acute coronary syndrome is suspected in most cases. In these patients, ECMO can be supportive in performing in situ thrombolysis or surgical thrombectomy [312,313].

However, hypoxic brain damage immediately following ECMO can result in a poor neurologic outcome. Therefore, brain CT just after ECMO and follow-up brain imaging may help in predicting neurologic outcomes and survival [311]. Additionally, intracranial hemorrhage (ICH) is a common complication in adults treated with ECMO and is associated with increased mortality. Treating ICH during ECMO represents a balance between pro- and anticoagulatory demands. Neurosurgical treatment can be successful in selected cases [314]. Therefore, if an ICH is suspected, a cerebral CT scan must be the priority over any subsequent interventions or ECMO insertion [315].

WLST

WLST because of perceived neurological injury and assumed poor prognosis (WLST-N) within the first 72 hours of hospitalization is common after OHCA and is associated with approximately one-third to 41% of in-hospital mortality overall, and 26% in TTM-treated OHCA survivors [95,151]. Considering TTM measures, their application is not the only factor influencing mortality as a result of WLST. TTM measures have also been shown as a factor with an influence on ICU admission to WLST time (7.6 days in patients receiving TTM treatment vs. 1.6 days in patients without TTM measures applied) [316,317].

Patients exposed to WLST-N within the first 72 hours of hospitalization resemble unexposed patients, with approximately a 26% chance of survival. Of these, more than 60% are predicted to have survival with favorable functional status. A study published in 2016 emphasized that failure to control the effects of WLST-N on mortality in post-OHCA patients may significantly bias the results of studies of OHCA or other severe brain injuries. The same study also suggested that reducing WLST-N within the first 72 hours of hospitalization may have important public health implications and that it may be an opportunity to decrease mortality after OHCA [154].

High mortality rates occurring due to WLST, however, are constantly emphasizing the need for reassessing the indications for WLST, to avoid inappropriate withdrawal of medical care which may result in unnecessary loss of life, given the fact that in-hospital mortality of these patients is very high even when not taking into consideration these treatment limitation decisions [318,319]. American Heart Association recommendations published in 2015 suggesting that neuro-prognostication should be carried out at least 72 hours after the completion of post-TTM rewarming and at least 72 hours after ROSC in patients not receiving TTM [165] are recently adjusted recommending that observation should be prolonged to seven days after the end of TTM or sedation suspension [171,320]. Also, there are suggestions that the observation period should be prolonged in males, patients with pre-hospital ROSC, patients receiving TTM, and patients who received extracorporeal CPR. If a seven-day EEG demonstrates an unfavorable pattern, it is unlikely that a longer observation period would be beneficial [118].

## Conclusions

As one of the main direct causes of mortality globally, OHCA is still a very important public health issue. Although post-admission measures are crucial in increasing survival and recovery chances in initial OHCA survivors, it is necessary to keep a constant focus on pre-admission steps to ensure ICU ward admission of patients with ROSC. Since most OHCA patients have underlying ischemic heart disease, urgent coronary reperfusion should be imperative in the palette of the first post-admission steps, followed by an initial assessment of neurological status and initial prediction of neurological prognosis. Preventing prolonged ischemia-reperfusion injury of all organs, lowering metabolic demands, and keeping perfusion at a satisfactory level, by maintaining MAP, oxygen, and carbon dioxide levels within the limits, may ensure adequate functioning of all important tissues during critical recovery period. Diaphragm function monitoring is also crucial for complete recovery and future research on monitoring diaphragm function in the ICU should focus on investigating the correlation between its standard markers and determinants and new ultrasonography-based indices, as well as electrical activity. Also, standardization in the form of specific good practice guidelines for diaphragm ultrasound and electrical activity recording in mechanically ventilated patients is necessary to optimize this specific function in hospitalized patients. Additionally, neuroprognostication is one of the most important aspects in the management of CA survivors. Proper strategy in multimodal neurological assessment approach could reduce WLST-N occurrence frequency and improve survival in these patients.

Since there is a paucity of data in post-CA patients regarding application of TTM measures, there is a large need for further investigations in this field. Also, awareness of the impact of WLST-N on outcomes may help guide providers and families away from early limitations in medical care based on perceived neurological prognosis. Lastly, management of CA survivors is achieved in the most comprehensive way in high-frequency post-resuscitation ICUs, where enteral nutrition is favored over parenteral nutrition and where renal and vasopressor support is rarely initiated. With adequate and organized application of all these measures, survival and successful recovery of post-CA patients can be brought to the highest possible level.
